# Attitudes to proposed assessment of pharmacy skills in Korean pharmacist licensure examination

**DOI:** 10.3352/jeehp.2017.14.6

**Published:** 2017-03-27

**Authors:** Joo Hee Kim, Ju-Yeun Lee, Young Sook Lee, Chul-Soon Yong, Nayoung Han, Hye Sun Gwak, Jungmi Oh, Byung Koo Lee, Sukhyang Lee

**Affiliations:** 1 College of Pharmacy & Institute of Pharmaceutical Science and Technology, Ajou University, Suwon, Korea; 2 College of Pharmacy & Division of Life and Pharmaceutical Sciences, Ewha Womans University, Seoul, Korea; 3 College of Pharmacy & Institute of Pharmaceutical Science and Technology, Hanyang University, Ansan, Korea; 4 College of Pharmacy, Keimyung University, Daegu, Korea; 5 College of Pharmacy, Yeungnam University, Gyeongsan, Korea; 6 College of Pharmacy, Chungnam National University, Deajeon, Korea; 7 College of Pharmacy, Seoul National University, Seoul, Korea; 8 Korean Pharmaceutical Information Center, Seoul, Korea; Hallym University, Korea

**Keywords:** Korean pharmacist licensure exam, Pharmacy skills assessment, Survey

## Abstract

**Purpose:**

The survey aimed to obtain opinions about a proposed implementation of pharmacy skills assessment in Korean pharmacist licensure examination (KPLE).

**Methods:**

A 16-question survey was distributed electronically to 2,738 people including 570 pharmacy professors of 35 pharmacy schools, 550 preceptors from 865 practice sites and 1,618 students who graduated in 2015. The survey solicited responses concerning the adequacy of the current KPLE in assessing pharmacy knowledge/skills/attitudes, deficiencies of pharmacy skills testing in assessing the professional competencies necessary for pharmacists, plans for pharmacy skills tests in the current KPLE, and subject areas of pharmacy practice.

**Results:**

A total of 466 surveys were returned. The current exam is not adequate for assessing skills and attitudes according to 42%–48% of respondents. Sixty percent felt that skills test is necessary to assess qualifications and professional competencies. Almost two-thirds of participants stated that testing should be implemented within 5 years. More than 60% agreed that candidates should be graduates and that written and skills test scores can be combined for pass-fail decisions. About 70% of respondents felt that the test should be less than 2 hours in duration. Over half of the respondents thought that the assessor should be a pharmacy faculty member with at least 5 years of clinical experience. Up to 70% stated that activities related to patient care were appropriate and practical for the scope of skills test.

**Conclusion:**

Pharmacy skills assessment was supported by the majority of respondents.

## Introduction

Traditional perception of pharmacy as an occupation is to promote and support the safe and rational use of medicines. Over the past several decades this view has shifted in many countries, with pharmacists regarded as a care-giver rather than a dispenser of medications. Increasingly, attention has been focused on pharmaceutical care skills and professional attitudes, rather than on pharmaceutical knowledge that is germane in the preparation of medical products. These patient-focused activities have evolved to the concept of pharmaceutical care, which is defined as the responsible provision of drug therapy that aims to improve a patient’s quality of life [1]. This concept of pharmaceutical care that encompasses the pharmacist as a care-giver, decision-maker, communicator, leader, manager, life-long learner, and teacher was embodied in 1997 by the World Health Organization (WHO) as the framework of the ‘seven-star pharmacist’ concept [2].

Korea followed the lead of WHO in 2011 in response to the needs of patient-centered professionalism by instituting a new 6-year pharmacy educational system that includes a 1-year of clinical clerkship. In this system, the mandatory duration of pharmacy practice experience is 35 weeks (1,400 hours) for 30 credits, which includes fulltime in-service training in a community pharmacy, a hospital pharmacy, a pharmaceutical industry, and pharmaceutical policy and management organization. The Korean pharmacist licensure examination was restructured in 2015 to reflect the changes in the 6-year pharmacy curriculum. Questions on clinical and practical pharmacy take the largest portion (30%, 104 questions) of the total of 350 questions, followed by biopharmacy (28.6%, 100 questions), industrial pharmacy (25.7%, 90 questions), and pharmacy legislation (5.7%, 20 questions) [3]. All questions are in multiple-choice format with 325 minutes of test time allocated in one day with no performancebased tests.

Assessment tests for clinical skills and attitudes that have been widely adopted in various settings in other countries include certification examination and maintenance of competency reviews. In 2000, the Pharmacy Examination Board of Canada adopted the objective structured clinical examination (OSCE) as a component of the countries’ performance-based pharmacist licensure examination [4]. In Japan, fourth-year pharmacy students are required to pass the ‘pharmaceutical common achievement test,’ which is a combination of computer-based testing and OSCE before attending the pharmacy clerkship [5]. To register as a pharmacist within the United Kingdom, a candidate is required to successfully complete a 52-week preregistration training to meet performance standards of personal effectiveness, interpersonal skills, medicine, and health, which are continuously monitored and assessed in the workplace by the tutor or trainer [6].

In 2009, the clinical skills test (CST) was adopted in the Korean medical licensure examination (KMLE) as a valid and reliable method to evaluate the clinical competency of medical graduates [7]. In addition, the Korean National Health Personnel Licensure Examination Board (KNHPLEB) recently announced that the CST would be implemented in the near future as a component of Korean Dental Licensure Examination on the basis of its success in the medical licensure examination. Similarly, implementing CST to the Korean pharmacist licensure examination is under consideration by KNHPLEB.

This study sought general opinions of the important stakeholders of pharmacy education concerning the needs and validity of pharmacy skills assessment for the Korean pharmacist licensure examination.

## Methods

The online survey using multiple-choice and short-answer questions was conducted via Google Docs between August 1 and August 31, 2015. All 35 schools of pharmacy accredited through the Korean Association of Pharmacy Education, the Korean Society of Health system Pharmacists, and the Korea Pharmaceutical Association were contacted by conventional or electronic mail and asked to complete the survey. Those contacted included 570 pharmacy professors, 1,618 graduates of the 6-year pharmacy program and 550 preceptors from 865 practice sites. Personal information including age, gender, profession, period of current service, and the geographical regions were collected and held strictly under the terms of the Korea Personal Information Protection Act. This study was reviewed and approved by the institutional review board of Ewha Womans University (IRB No. 99-4).

A 16-item questionnaire was developed to measure the opinions about four topics (Table 1). The first topic was the adequacy of current pharmacist licensure examination in assessing pharmacy knowledge/skills/attitudes. The second topic was the needs and validity of pharmacy skills test in assessing the qualifications and professional competencies needed for pharmacists. The third topic was the implementation plan of pharmacy skills tests in current pharmacist licensure examination. The fourth topic was the subject areas of pharmacy practice included on the pharmacy skills test. The 5-point Likert scale (‘strongly disagree,’ ‘disagree,’ ‘neutral,’ ‘agree,’ or ‘strongly agree’) and nominal scales were used for the first three topics. The appropriateness and practicality of each subject area for the skills tests were surveyed and analyzed. The core subject areas on skills test formulated through the job analysis of the Korean Association of Pharmacy Education were pharmacy practice, hospital pharmacy, community pharmacy, industrial pharmacy and others [8].

The survey data were analyzed using SAS Enterprise Guide ver. 6.1 software (SAS Institute Inc., Cary, NC, USA). The proportion of respondents answering for each item of questions 1 to 16 and the mean scores of Likert Scale (1 to 5) of questions 1 to 5 were measured for statistical analysis. The analysis of variance analysis was used to test the response scores from questions 1 to 5 and the Pearson chisquare analysis was used to test statistical differences in proportions of the data from questions 6 to 11. With Cronbach’s alpha analysis, the internal consistency reliabilities for each of the scales of questionnaires were measured.

## Results

The 466 survey respondents were evenly distributed between trainers (professors and preceptors) and trainees (pharmacy graduates) (Table 2). Approximately 64% of the overall participants were female. About two-thirds of the professors were male and about 87% of the preceptors were female. About 54% of the professors had less than 5 years of work experience and about 69% of preceptors had less than 15 years of work experience.

**Fig. 1.**
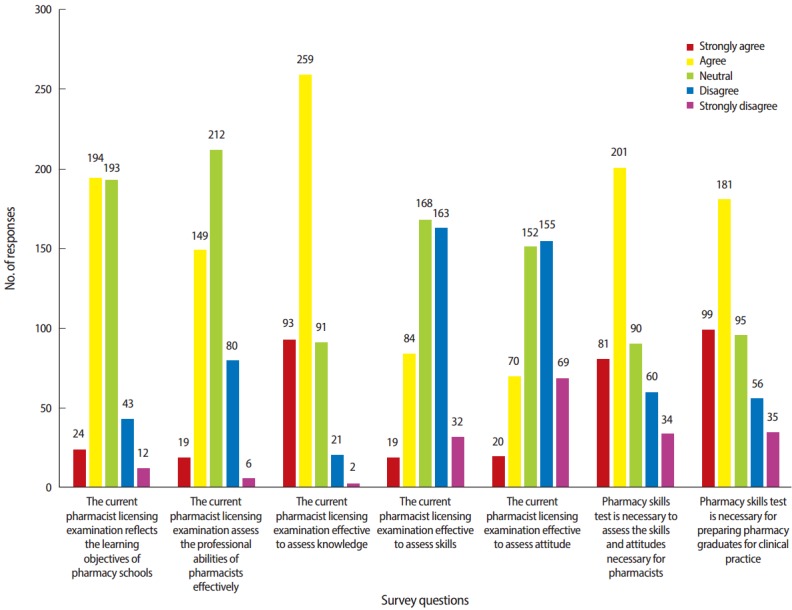
Survey results about adequacy of the current pharmacist licensure examination and needs of pharmacy skills test.

Survey results of questions 1 to 5 concerning the adequacy of current pharmacist licensure examination in assessing pharmacy knowledge/skills/attitudes and the needs and validity of pharmacy skills test in assessing the qualifications and professional competencies necessary for pharmacists are shown in Fig. 1. Fifty five (11.8%) of participants disagreed that the current pharmacist licensure examination reflects the learning objectives of pharmacy schools.

Eighty six (18.5%) respondents disagreed that the current pharmacist licensure examination assesses the professional abilities of pharmacist effectively; 42 (48.8%) of the respondents were pharmacy graduates. Three hundred and fifty-two (75.5%) of the respondents agreed that the current exam effectively assesses pharmaceutical knowledge, but 195 (41.8%) and 224 (48.1%) answered that the current exam does not effectively assess the skills and attitudes of the pharmacy profession, respectively. In addition, 89 (45.6%) of the 195 respondents and 89 (39.7%) of the 224 respondents were pharmacy graduates. Two hundred and eighty-two (60.5%) agreed that pharmacy skills test is necessary to assess the skills and attitudes and 280 (60.1%) agreed that it is necessary to prepare the pharmacy graduates for clinical practice. One hundred and thirty-four (47.5%) out of the 282 respondents and 137 (48.9%) of the 280 respondents were pharmacy graduates.

The types of professions of the respondents and the length of their work experience were correlated with these attitudes toward the adequacy of current licensure examination in terms of reflecting learning objectives of pharmacy schools and assessing professional abilities of pharmacists, and pharmaceutical knowledge (P<0.05). The attitudes to the necessity and validity of pharmacy skills test in assessing the qualifications and professional competencies necessary for pharmacist were also related to the types of professions and the length of their work experience (P< 0.05).

**Fig. 2.**
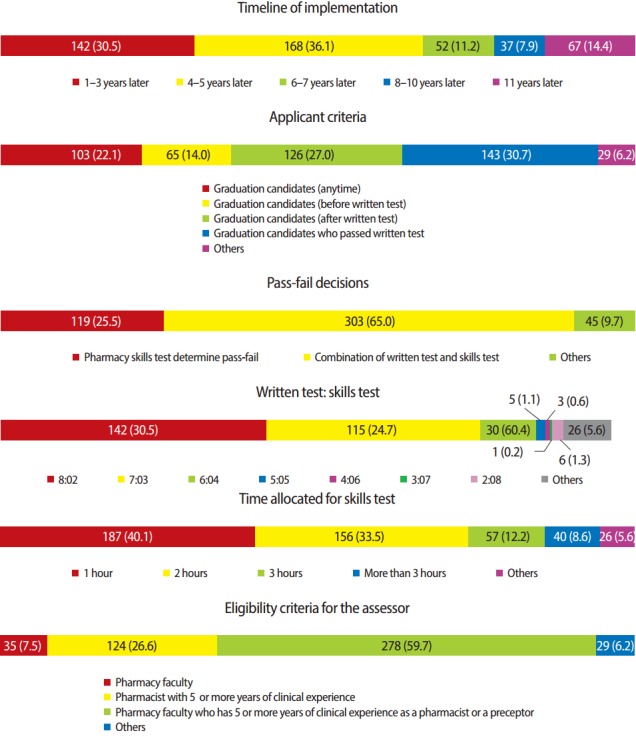
Survey results about the implementation plan for pharmacy skills test. Values are presented as number (%).

Survey results concerning the implementation plan for pharmacy skills testing are presented in Fig. 2. Twenty two (4.7%) respondents disapproved of the implementation of pharmacy skills test in the Korean pharmacist licensure examination. Concerning the timeline for the implementation of pharmacy skills test in the current examination, 168 (36.1%) participants thought that the appropriate timeline is within 4–5 years and 142 (30.5%) participants thought that it would be appropriate to implement the test within 1–3 years. Forty one (33.3%) of 123 professors thought that a much longer implementation period of 11 years is appropriate, with 45.0% of 129 preceptors preferring a shorter period of 4–5 years and 46.6% of 193 pharmacy graduates selecting 1–3 years. One hundred and forty-three (30.7%) thought that graduation candidates who pass the written test should take a pharmacy skills test, and 294 (63%) agreed that the criteria of the written and skills tests should be the same. Three hundred and three (65%) respondents opinioned that student scores of the pharmacy skills and written tests should be combined for ‘passfail’ decisions in the pharmacist licensure examination. In addition, 153 (46.2%) and 113 (34.7%) participants noted that the ratios of the two tests should be 8:2 and 7:3, respectively. One hundred and eighty-seven (40%) and 156 (33.5%) participants thought that 1- and 2-hour pharmacy skills test would be appropriate, respectively. Over a third of professors, preceptors, and pharmacy graduates agreed that 1- or 2-hour skills test would be appropriate. Two hundred and seventy-eight (59.7%) of participants stated that pharmacy faculty with 5 or more years of clinical experience as a pharmacist or a preceptor would be the appropriate eligibility criteria for an assessor. The types of professions of the respondents and the length of their work experience showed no correlation with their preferences regarding the implementation plan for pharmacy skills test in current pharmacist licensure examination.

The survey results concerning the material to be included in a pharmacy practice skills test are presented in Table 3. More than 70% of all respondents felt that the test needs to examine patient reception, filing prescriptions, dispensing prescriptions, patient counseling, drug information, and safety management. Pharmacist activities relating to hospital pharmacy and community pharmacy were considered appropriate and practical by 50%–70% of respondents. Activities related to industrial pharmacy were considered appropriate by 53% of participants and practical by 42% of participants. Drug development, drug distribution, education, and research, which were not included in the Advanced Pharmacy Practice Experience program of Korean pharmacy schools, were considered appropriate and practical by less than 34% of participants.

## Discussion

The purpose of a new 6-year pharmacy educational system in Korea was to provide pharmacy students with broad opportunities to perform various learning activities in real-life clinical settings and to improve their competency in pharmacy practice. Currently, evaluation of students in clinical clerkships is accomplished through a combination of direct observation of clinical performance, case report presentation, and/or written examination. The individual preceptor is usually responsible for providing a written evaluation of student’s performance, which is forwarded to pharmacy professors for a final grade of clerkship. Therefore, the student’s clinical competence is currently evaluated by subjective appraisal of skills and attitudes by the preceptor, which may not correlate with pharmaceutical knowledge.

To be licensed as a pharmacist in Korea, a candidate must complete an approved pharmacy education program including clerkship and pass all four parts of pharmacist licensure examination given by KNHPLEB. The pharmacist licensure examination aims to determine if a candidate has sufficient knowledge in all areas of pharmaceutical education curricula in Korea. In addition, a current exam consisting of multiple-choice questions has limited ability to evaluate the behavioral competency of candidates as well as their fitness to practice [9]. The emphasis on patient-oriented care in the modern health care system necessitates that reliable and valid assessment methods are used for summative evaluation of pharmacist candidates.

This study provides important insights into the implementation of pharmacy skills assessment in the current pharmacist licensure examination in Korea. There was a realization among the survey respondents that current licensure examination formats may not adequately measure some important competencies needed for entry-level pharmacists. About a half of the respondents disagreed that the current exam effectively assesses the skills and attitudes of pharmacist candidates and over 60% stated that a skills test is essential in nurturing competent pharmacists for their clinical disciplines. According to two-thirds of the respondents (mostly preceptors and pharmacy graduates), the skills test can be implemented within less than 5 years. Up to 70% of the respondents felt that pharmacist activities related to patient care including reception, counseling and dispensing are appropriate as well as practical for the pharmacy skills test.

Well-designed CSTs have established reliability and validity in pharmacy and other health professions. In Canada, the comprehensive information and data collected through extensive consultation with stakeholders, development of a blueprint outlining competencies to be assessed, validation of multiple OSCE stations, field testing of stations and protocols for data collection and analysis eventually confirmed that the OSCE can be used for competency assessment in pharmacy [10]. Recent studies have demonstrated that the introduction of CST to KMLE improved the clinical performance of medical graduates as well as the quality of medical education in Korea [11].

Significant planning, coordination of multiple resources, commitment to testing, and judicious use of assessment data would be necessary for successful implementation of pharmacy skills test. In addition, broader and various aspects of performance-based tests may be surveyed to validate the needs and feasibilities of pharmacy skills tests before its implementation. Each school of pharmacy should judge the relative value of pharmacy skills test in light of local resources as well as the need to prepare students for the licensure examination. Moreover, it would be essential to find validity and reliability evidence to justify the use of pharmacy skills test in order to minimize the multiple potential sources of error before launching the test.

In conclusion, advocates of pharmacy skills test should continue to produce and disseminate evidence for the impact that is witnessed by pharmacy stakeholders including the general public.

## Figures and Tables

**Table 1.** The survey questionnaire for the pharmacist licensure examination

Topic	16-item questionnaire
Adequacy of current pharmacist licensing examination in assessing pharmacy knowledge/skills/attitudes	1. Do you think that the current pharmacist licensure examination reflects the learning objectives of pharmacy schools?
	2. Do you think that the current pharmacist licensure examination assesses the professional abilities of pharmacists effectively? effectively?
	3. Do you think that the current pharmacist licensure examination effective to assess ⑴ knowledge, ⑵ skills, and (3) attitude?
Needs and validity of pharmacy skills test in assessing the qualifications and professional competencies necessary for pharmacists	4. Do you think that pharmacy skills test is necessary to assess the skills and attitudes necessary for pharmacists?
	5. Do you think that pharmacy skills test is necessary for preparing pharmacy graduates for clinical practice?
Implementation plan for pharmacy skills tests in current pharmacist licensure examination	6. When do you think is the most appropriate to adopt pharmacy skills test?
	7. What do you think is the most appropriate criteria for applicant for pharmacy skills test?
	8. What do you think is the most appropriate criteria for pass-fail decision?
	9. What do you think is the most appropriate proportions of skills test in pharmacist licensing exam?
	10. What do you think is the most appropriate time allocated for pharmacy skills test?
	11. What do you think is the most appropriate eligibility criteria for assessor of pharmacy skills test?
Subjects of pharmacy practice that are included in the skills test (appropriateness and practicality)	12. Pharmacy practice (patient reception, filing prescription, dispensing, patient counseling, drug information, safety management)
	13. Hospital pharmacy (patient care, clinical study management)
	14. Community pharmacy (pharmacy management, non-prescription product distribution)
	15. Industrial pharmacy (pharmaceutics management, product quality management)
	16. Others (drug development, drug distribution, education and research, public health management)

**Table 2.** Participant characteristics of survey about the pharmacist licensure examination

Characteristic	Total (n = 466)	Professors (n = 123)	Preceptors (n = 129)	Graduates (n = 193)	No answers (n = 21)
Gender					
Men	166 (35.6)	76 (61.8)	17 (13.2)	63 (32.6)	10 (47.6)
Women	300 (64.4)	47 (38.2)	112 (86.8)	130 (67.4)	11 (52.4)
Age (yr)					
20-29	155 (33.3)	1 (0.8)	17 (13.2)	135 (69.9)	2 (9.5)
30-39	140 (30.0)	23 (18.7)	61 (47.3)	55 (28.5)	1 (4.8)
40-49	102 (21.9)	55 (44.7)	32 (24.8)	3 (1.6)	12 (57.1)
50-59	55 (11.8)	34 (27.6)	16 (12.4)	0	5 (23.8)
≥60	14 (3.0)	10 (8.1)	3 (2.3)	0	1 (4.8)
Years of working experience					
<5	288 (61.8)	66 (53.7)	21 (16.3)	193 (100)	8 (38.1)
5-9	49 (10.5)	13 (10.6)	33 (25.6)	0	3 (14.3)
10-14	54 (11.6)	17 (13.8)	35 (27.1)	0	2 (9.5)
15-19	19 (4.1)	6 (4.9)	11 (8.5)	0	2 (9.5)
>20	56 (12.0)	21 (17.1)	29 (22.5)	0	6 (28.6)
Regions					
Seoul/Gyeonggi	271 (58.2)	63 (51.2)	81 (62.8)	112 (58)	15 (71.4)
Gangwon	4 (0.9)	2 (1.6)	0	2 (1)	0
Chungcheong	28 (6.0)	13 (10.6)	1 (0.8)	11 (5.7)	3 (14.3)
Jeonra	29 (6.2)	19 (15.4)	0	8 (4.1)	2 (9.5)
Gyeongsang	134 (28.8)	26 (21.1)	47 (36.4)	60 (31.1)	1 (4.8)

Values are presented as number (%).

**Table 3.** Survey results about scope of pharmacy practice for the pharmacy skills test

Subject area	Appropriateness		Practicality	
	Yes	No	Yes	No
Pharmacy practice				
Patient reception	356 (76.4)	110 (23.6)	333 (71.5)	133 (28.5)
Filing prescription	405 (86.9)	61 (13.1)	397 (85.2)	69 (14.8)
Dispensing	399 (85.6)	67 (14.4)	368 (79.0)	98 (21.0)
Patient counseling	426 (91.4)	40 (8.6)	407 (87.3)	59 (12.7)
Drug information	408 (87.6)	58 (12.4)	389 (83.5)	77 (16.5)
Safety management	370 (79.4)	96 (20.6)	345 (74.0)	121 (26.0)
Hospital pharmacy				
Patient care	325 (69.7)	141 (30.3)	303 (65.0)	163 (35.0)
Clinical study management	246 (52.8)	220 (47.2)	233 (50.0)	233 (50.0)
Community pharmacy				
Pharmacy management	312 (67.0)	154 (33.0)	276 (59.2)	190 (40.8)
Non-prescription product distribution	321 (68.9)	145 (31.1)	305 (65.5)	161 (34.5)
Industrial pharmacy				
Pharmaceutics management	246 (52.8)	220 (47.2)	190 (40.8)	276 (59.2)
Product quality management	251 (53.9)	215 (46.1)	195 (41.8)	271 (58.2)
Others				
Drug development	123 (26.4)	343 (73.6)	123 (26.4)	343 (73.6)
Drug distribution	146 (31.3)	320 (68.7)	133 (28.5)	333 (71.5)
Education and research	160 (34.3)	306 (65.7)	159 (34.1)	307 (65.9)
Public health management	255 (54.7)	211 (45.3)	247 (53.0)	219 (47.0)

Values are presented as number (%).

## Supplementary material

Supplement 1. Data file is available from https://dataverse.harvard.edu/dataset.xhtml?persistentId=doi:10.7910/DVN/XSF8XW

Supplement 2. Audio recording of the abstract.
